# Association Between Antibiotic Prophylaxis Before Cystectomy or Stent Removal and Infection Complications: A Systematic Review

**DOI:** 10.1016/j.euf.2023.01.012

**Published:** 2023-01-27

**Authors:** Luca Antonelli, Kirby Sebro, Abdelilah Lahmar, Peter C. Black, Saum Ghodoussipour, Jill M. Hamilton-Reeves, Jay Shah, Jensen Bente Thoft, Seth Paul Lerner, Carlos Llorente, Ilaria Lucca, Mark A. Preston, Sarah P. Psutka, John P. Sfakianos, Susanne Vahr Lauridsen, Stephen B. Williams, James Catto, Hooman Djaladat, Wassim Kassouf, Katherine Loftus, Siamak Daneshmand, Christian D. Fankhauser

**Affiliations:** a Department of Urology, Luzerner Kantonsspital, University of Lucerne, Lucerne, Switzerland; b Department of Urology, Policlinico Umberto I, Sapienza University, Rome, Italy; c Department of Urology, Western General Hospital, Edinburgh, UK; d Medicine, Faculty of Medicine and Pharmacy, Mohammed VI University Hospital, Oujda, Morocco; e Vancouver Prostate Centre and Department of Urologic Sciences, University of British Columbia, Vancouver, BC, Canada; f Section of Urologic Oncology, Rutgers Cancer Institute of New Jersey, New Brunswick, NJ, USA; g Department of Urology, University of Kansas Medical Center, Kansas City, KS, USA; h Department of Urology, Stanford University School of Medicine, Stanford, CA, USA; i Department of Urology, Aarhus University Hospital, Aarhus, Denmark; j Scott Department of Urology, Dan L. Duncan Cancer Center, Baylor College of Medicine, Houston, TX, USA; k Department of Urology and Research Unit, Hospital Universitario Fundación Alcorcon, Alcorcón, Madrid, Spain; l Department of Urology, CHUV, Lausanne, Switzerland; m Division of Urological Surgery and Center for Surgery and Public Health, Brigham and Women’s Hospital, Harvard Medical School, Boston, MA, USA; n Department of Urology, University of Washington, Seattle, WA, USA; o Department of Urology, Icahn School of Medicine at Mount Sinai, New York, NY, USA; p Department of Urology, Copenhagen University Hospital, Copenhagen, Denmark; q WHO-CC, Parker Institute Bispebjerg & Frederiksberg University Hospital, Copenhagen, Denmark; r Division of Urology, Department of Surgery, University of Texas Medical Branch, Galveston, TX, USA; s Academic Urology Unit, University of Sheffield, Sheffield, UK; t Institute of Urology, Kenneth Norris Jr. Comprehensive Cancer Center, University of Southern California, Los Angeles, CA, USA; u Department of Surgery (Urology), Faculty of Medicine, McGill University, Montreal, Quebec, Canada; v Department of Anesthesiology, Perioperative and Pain Medicine, Icahn school of Medicine at Mount Sinai, New York, NY, USA; w Department of Urology, Keck School of Medicine, University of Southern California, Los Angeles, CA, USA

**Keywords:** Cystectomy, Infection, Postoperative complications, Antibiotic prophylaxis, Sepsis

## Abstract

**Context:**

Patients undergoing radical cystectomy frequently suffer from infectious complications, including urinary tract infections (UTIs) and surgical site infections (SSIs) leading to emergency department visits, hospital readmission, and added cost.

**Objective:**

To summarize the literature regarding perioperative antibiotic prophylaxis, ureteric stent usage, and prevalence of infectious complications after cystectomy.

**Evidence acquisition:**

**A s**ystematic review of PubMed/Medline, EMBASE, Cochrane Library, and reference lists was conducted.

**Evidence synthesis:**

We identified 20 reports including a total of 55 306 patients. The median rates of any infection, UTIs, SSIs, and bacteremia were 40%, 20%, 11%, and 6%, respectively. Perioperative antibiotic prophylaxis differed substantially between reports. Perioperative antibiotics were used only during surgery in one study but were continued over several days after surgery in all other studies. Empirical use of antibiotics for 1–3 d after surgery was described in 12 studies, 3–10 d in two studies, and >10 d in four studies. Time to stent removal ranged from 4 to 25 d after cystectomy. Prophylactic antibiotics were used before stent removal in nine of 20 studies; two of these studies used targeted antibiotics based on urine cultures from the ureteric stents, and the other seven studies used a single shot or 2 d of empirical antibiotics. Studies with any prophylactic antibiotic before stent removal found a lower median percentage of positive blood cultures after stent removal than studies without prophylactic antibiotics before stent removal (2% vs 9%).

**Conclusions:**

We confirmed a high proportion of infectious complications after cystectomy, and a heterogeneous pattern of choice and duration of antibiotics during and after surgery or stent removal. These findings highlight a need for further studies and support quality prospective trials.

**Patient summary:**

In this review, we observed wide variability in the use of antibiotics before or after surgical removal of the bladder.

## 1. Introduction

Cystectomy with urinary diversion is a common treatment option for patients with pelvic cancers or bladder dysfunction, but it is associated with potentially life-threatening complications and 90-d mortality of 4.7% (range 0.0–7.0%) [1,2]. Within the first 30 d after surgery, infections contribute significantly to postoperative morbidity [2–4], and bacterial sources include spillage of urine from the urinary tract or bowel, which is frequently used for urinary diversion. After the minor trauma caused by removal of the ureteric stents, a second peak of infectious complications can be observed. Therefore, perioperative empirical antibiotic prophylaxis should be considered to decrease the risk of infectious complications after cystectomy or stent removal. However, the evidence to support this approach is limited, and most recommendations have been derived from colorectal procedures [5]. The aim of this review is to summarize the published literature regarding the impact of antibiotic prophylaxis in the perioperative period and at the time of stent removal on the prevalence and severity of infectious complications after cystectomy.

## 2. Evidence acquisition

In accordance with the Preferred Reporting Items for Systematic Reviews and Meta-analyses (PRISMA) statement [6], a systematic literature search was conducted on November 29, 2021, using the electronic databases PubMed, EMBASE, and Cochrane Library. Several keyword combinations, synonyms, and search terms were used to identify sources related to infectious complications and cystectomy. The detailed search protocol can be found in the Supplementary material.

Articles were screened in a two-stage selection process using Covidence (Veritas Health Innovation Ltd 2016; LLC, 520 Lake Cook Road, Suite 350, Deerfield, IL, 60015). In the first stage, three authors (V.P., K.S., and A.L.) reviewed the abstracts; animal series and non-English studies were excluded unless the corresponding abstracts were available in English or the full texts were available in French, Spanish, Italian, or German. When in doubt, studies were maintained for further review. In the second stage, all titles, abstracts, and full texts were screened for appropriateness for inclusion by two investigators (K.S. and A.L.). Data from the same studies that appeared in multiple publications were considered only once. Any discrepancies were resolved by a third investigator (L.A.). Two independent reviewers assessed the methodological quality of each study, including the risk of bias and applicability, according to the Quality Assessment of Diagnostic Accuracy Studies-2 (QUADAS-2) [7]. QUADAS-2 describes the risk of bias and concerns regarding applicability of the studies based on four key domains: (1) patient selection, (2) index test, (3) reference standard, and (4) flow and timing. Study bias and applicability were evaluated as low, high, or unclear (Supplementary Table 1). Any disagreement was resolved by discussion, and the final decision was based on consensus.

Studies reporting on infectious complications after cystectomy and/or after the removal of ureteral stents, antibiotics used during and after cystectomy, and antibiotic prophylaxis before removal of ureteral stents were included in the review. Controls were not predefined in the search strategy to capture all relevant literature.

Data related to study design, patient inclusion criteria, major comorbidities, description of surgery, type and dose of antibiotics used during and after cystectomy and at removal of stents, and type and treatment of infectious complications were extracted from included studies. Two investigators (K.S. and A.L.) extracted the data, and a third investigator (L.A.) reviewed the extracted data. Disagreements were discussed and resolved by consensus.

Median percentages of any infectious complications, urinary tract infections (UTIs), surgical site infections (SSIs), and positive blood cultures in each report were calculated independently of sample size to illustrate the differences across the studies.

## 3. Evidence synthesis

After excluding duplicates, we identified 674 publications that met the initial search criteria and proceeded with a screening review of titles and abstracts for 46 full texts. Twenty studies were selected for inclusion, of which 15 were retrospective and five prospective (Supplementary Fig. 1 and Supplementary Table 2). The 20 studies included a total of 55 306 patients, 81% of whom were male and 19% female (the median number of patients per report: 102, interquartile range: 50–179).

A high risk of bias and/or applicability in patient selection was identified in studies that included only patients with malignant disease or excluded patients with specific allergies to antibiotics. The lack of a definition for infectious complications contributed to a high risk of bias and/or applicability for the index test. In some cases, the reference standard was not specified in the study, for example, regarding the choice to perform a urine culture before stent removal. A high risk of bias for flow and timing was identified in studies in which not all patients were followed postoperatively at the same center or readmission rates due to infectious complications were not available. No pooling/meta-analysis was possible because of the heterogeneity of the reports describing antibiotic prophylaxis for cystectomy and stent removal.

**Fig. 1 –:**
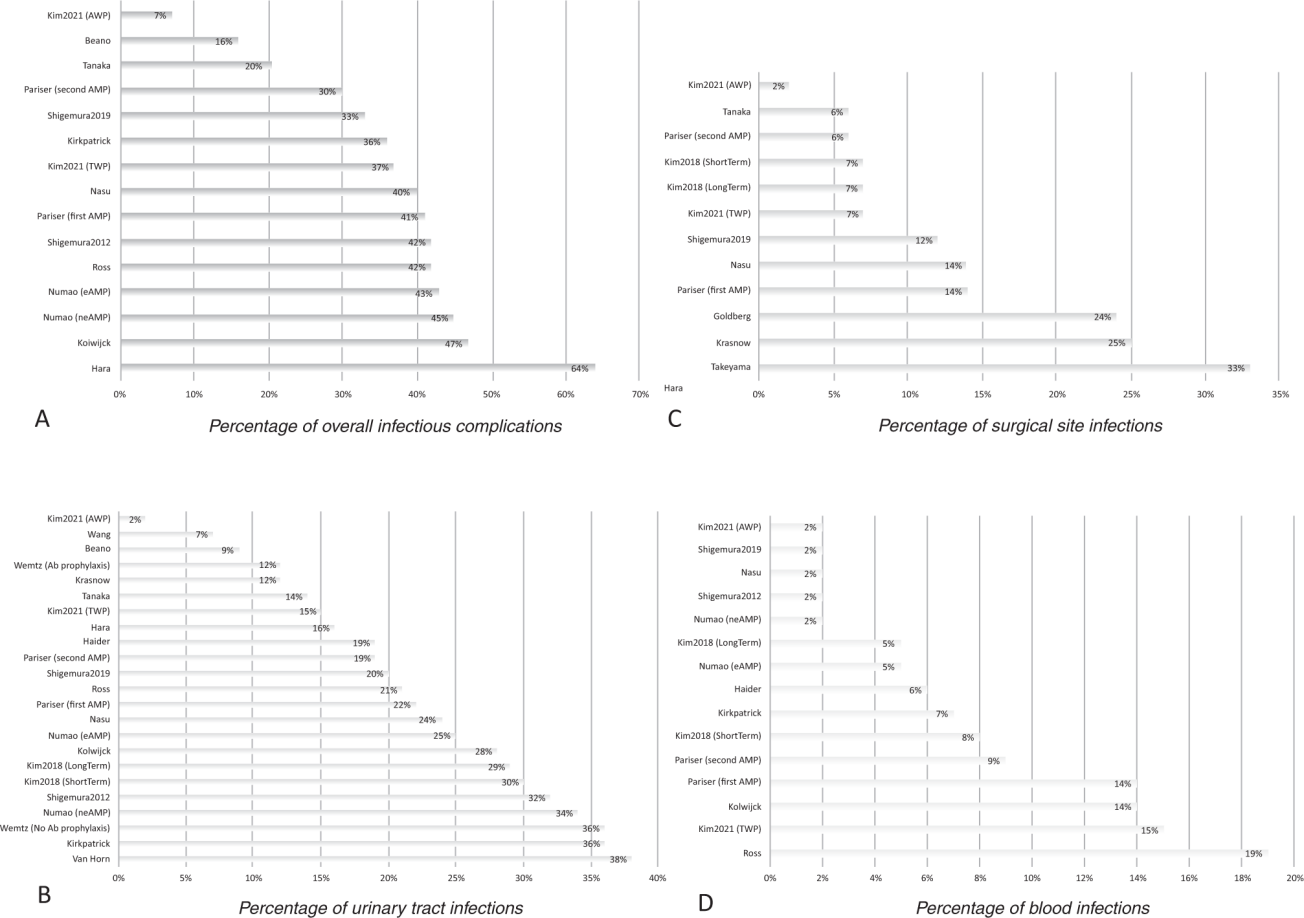
Infectious complications after cystectomy for each study: (A) any infectious complications, (B) urinary tract infections, (C) surgical site infections, and (D) blood infections. Ab = antibiotic; AWP = antibiotic-irrigating wound protector; eAMP – extended antimicrobial prophylaxis; neAMP = nonextended antimicrobial prophylaxis; TWP = traditional wound protector.

**Fig. 2 –:**
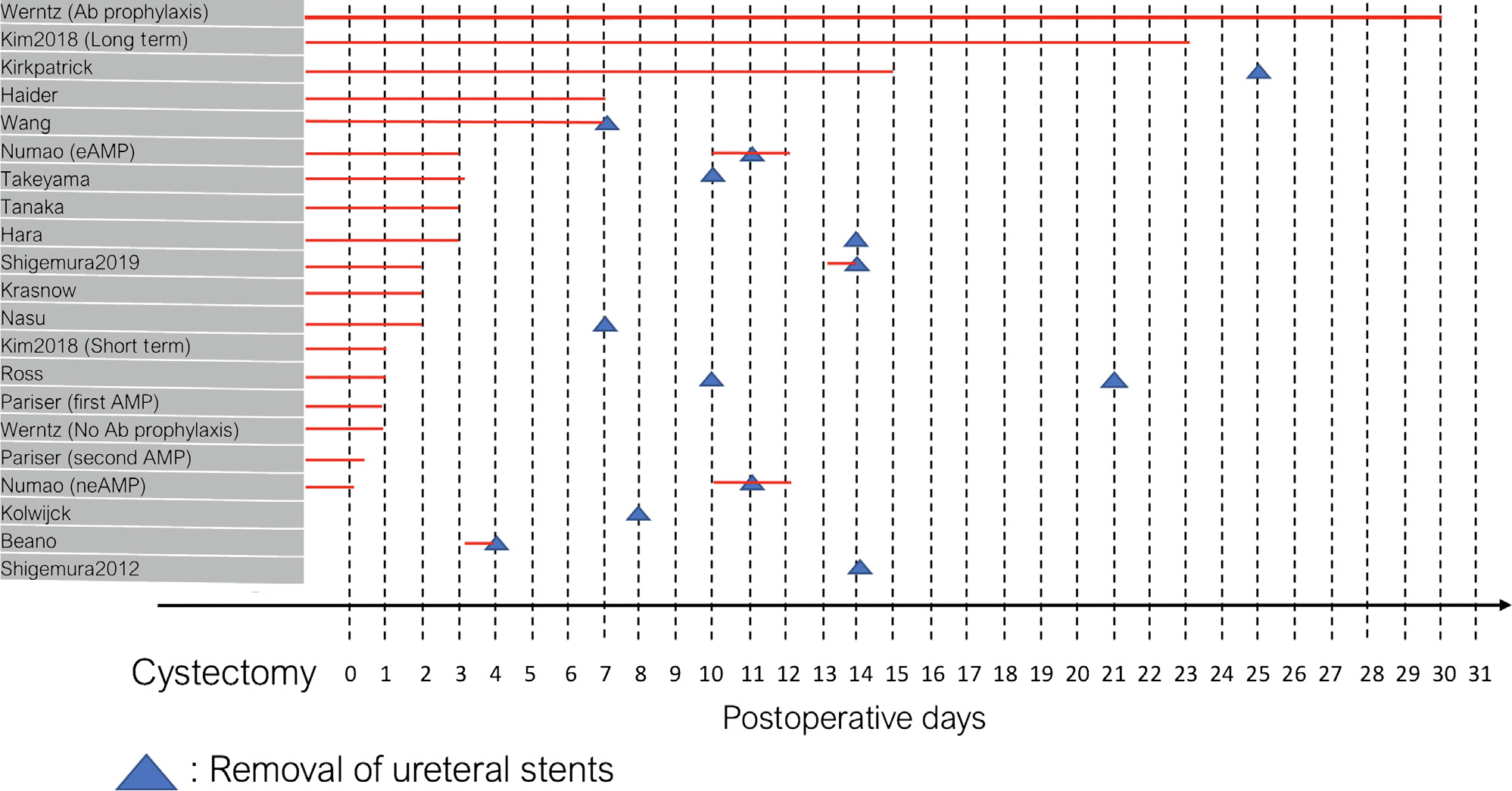
Length of antibiotic therapy administration (red line) at cystectomy and at stent removal.Ab = antibiotic; AMP = antimicrobial prophylaxis; eAMP = extended antimicrobial prophylaxis; neAMP = nonextended antimicrobial prophylaxis.

The proportions of any infections, UTIs, SSIs, and positive blood cultures were reported in 12, 18, 14, and 11 of 20 articles, respectively. The median percentages of any infections, UTIs, SSIs, and positive blood cultures were 40%, 20%, 11%, and 6%, respectively (Fig. 1). Perioperative antibiotic prophylaxis differed substantially between reports. One study reported antibiotic use before and during surgery only, whereas antibiotics were extended for 1–3 d after surgery in 12 studies [8–19], 3–10 d in two studies, and >10 d in four studies (Fig. 2) [10,18–23]. A detailed description of the perioperative antibiotic prophylaxis used can be found in the Supplementary material.

Other measures to prevent infections were described in two papers. Kim and colleagues [24] applied an antibiotic-irrigating wound protector (AWP) in 50% of patients, which resulted in a decreased percentage of overall infectious complications from 37% with a traditional wound protector to 7% with an AWP. Ross and colleagues [14] described a multipronged approach to reduce infections, including implementing a wound barrier, washing the wound with saline/povidone mixture following fascial closure, changing gowns and gloves prior to fascial closure, using a separate sterile closing tray, and applying an antimicrobial impregnated dressing.

The use of antibiotic prophylaxis before or during ureteral stent removal was described in nine of 20 papers. In studies using any prophylactic antibiotics, the median proportion of positive blood cultures was 2%, compared with 9% in studies without prophylactic antibiotics before stent removal. Beano [25] performed a urine culture from stents or urostomy bag before stent removal and administered a single-shot intravenous (IV) antibiotic based on the culture; they described UTIs in 70 of 146 patients (48%) after stent removal. Goldberg and colleagues [22] and Kim and colleagues [24] administered a single-shot IV third-generation cephalosporin before stent removal. Nasu [12] performed a urine culture and administered antibiotics based on the culture before stent removal, describing infectious complications in eight of 50 patients (16%) after stent removal. Numao and colleagues [8] administered daily oral dose of 500 mg levofloxacin for 2–3 d after stent removal, and additional antimicrobial agents were administered only if subsequent infection occurred (13% of patients). Shigemura and colleagues [15] used oral fluoroquinolones 1–2 h before stent removal in patients who had a positive urine culture before cystectomy (16/49) and described infectious complications in one of these 16 patients (6%), compared with six of 33 patients (18%) who did not receive an antibiotic prophylaxis for stent removal and had UTIs. Van Horn [26] administered single-shot aminoglycoside to 121 of 279 patients before stent removal and described subsequent UTIs in 30 of 279 patients (11%). Wang and colleagues [18] continued postoperative second-generation cephalosporin in 67 of 179 patients until stent removal and performed a urine culture before stent removal in 112 of 179 patients who received antibiotics for only 3 postoperative days. Werntz and colleagues [19] used an antibiotic prophylaxis at the time of stent removal for 50% of patients with a single shot of 160 mg/800 mg trimethoprim-sulfamethoxazole, 100 mg nitrofurantoin, or 250 mg ciprofloxacin. In 12 of 20 studies, researchers described the most common micro-organisms causing infections after cystectomy: *Enterococcus* in eight (67%), *Escherichia coli* in seven (58%), *Staphylococcus aureus* in seven of 12 (58%), *Klebsiella pneumoniae* in six (50%), and *Candida albicans* in four (33%) of 12 studies.

## 4. Conclusions

This review is, to our knowledge, the first reported systematic summary of the incidence of any infections, UTIs, SSIs, and positive blood cultures after cystectomy. The choice and duration of perioperative antibiotic prophylaxis varied substantially from intraoperative-only to empirical antibiotics during the first 30 postoperative days. The time of stent removal ranged from postoperative day 4 to 25, and the use and choice of antibiotic prophylaxis before stent removal were also heterogeneous.

The lack of consensus and high-quality studies regarding this topic prevents any strong recommendations or guidelines regarding potential interventions to decrease infectious complications following radical cystectomy. Currently, the American Urological Association (AUA) guidelines for antibiotic prophylaxis during urological surgeries recommend a single preoperative dose of cefazolin for cystectomies if the small bowel is used for urinary diversion [27] and antibiotic prophylaxis before bladder catheter removal in patients with risk factors but not before ureteral stent removal [5]. The current guidelines of the European Association of Urology do not comment on antibiotic prophylaxis during or after cystectomy or during stent removal [28].

Recent data suggest adherence to the current AUA guidelines as low as 30% [29], suggesting an important research niche to define the role of multiple potential interventions, including (1) obtaining a preoperative urine culture, (2) use of preoperative immunonutrition [30–32], (3) wound protectors or negative-pressure wound therapy [33–35], (4) choice and duration of empirical prophylactic antibiotics around cystectomy, (5) obtaining a urine culture using a correct technique before stent removal [36], (6) duration of stent usage and choice and duration of empirical prophylactic antibiotics around stent removal, and (7) bowel preparation [37,38]. These interventions may need to be personalized for individual patients based on risk factors for infectious complications, including diabetes, autoimmune diseases, poor nutrition, prior history of radiation, use of steroids, or surgical risks, including the type of urinary diversion [20]. Further, in the decision-making process regarding the type and dose of antibiotics used during and after cystectomy, it is important to consider that the prolonged use of antibiotic therapy may cause harm, including a potentially higher risk of resistant organism infectious complications [39], *Clostridium difficile* infection [40], or fungal infections. Another long-term side effect of the prolonged use of antibiotics is drug resistance, and antibiotics or bowel preparation may influence the efficacy of adjuvant immunotherapy [41].

According to meta-analyses of randomized trials, four simple recommendations decrease infectious complications after abdominal surgery, and these recommendations may also be applicable in cystectomy patients. First, gloves and instruments should be changed before abdominal wound closure [42]. Second, shaving of body hair should be avoided, and if hair has to be removed, clippers or depilatory cream should be preferred [43]. Third, adhesive drapes are not recommended [34]. Fourth, forced-air warming in the preoperative period or at least during surgery is encouraged [44].

Our review has several limitations. First, only 20 studies were identified, 15 of which were retrospective with a high risk of bias. It is possible that not all relevant studies were identified due to undetected imprecisions in the search strategy, which would be another potential source of bias. Second, there was heterogeneity in the description of the types and doses of antibiotic treatment and timing of stent removal among the eligible studies. Third, local resistance patterns may influence the efficacy of antibiotic prophylaxis. Fourth, the definition and description of infectious complications after cystectomy, especially UTIs, varied among the studies. Finally, the overall risk of bias was high among the studies.

In summary, the literature describing infectious complications after cystectomy is heterogeneous. The choice and duration of perioperative antibiotics differ substantially among series. Given this heterogeneity, we refrained from pooling the data in a meta-analysis. Nevertheless, the published literature describes a high proportion of infectious complications after cystectomy. Given the lack of consensus among guidelines on antibiotic prophylaxis during and after cystectomy and at the time of stent removal, there is a need for clinical trials in this setting.

## Supplementary Material

supplement 1

supplement 2

supplement 3

## Appendix A. Supplementary data

Supplementary data to this article can be found online at https://doi.org/10.1016/j.euf.2023.01.012.
