# Supplementary material for: Association Between Antibiotic Prophylaxis Before Cystectomy or Stent Removal and Infection Complications: A Systematic Review
Source: Eur Urol Focus. Author manuscript; Available in PMC 2026 Apr 13. (PMC13071900; doi:10.1016/j.euf.2023.01.012)
Supplement: supplement 1 [file NIHMS2153973-supplement-supplement_1.docx]

Supplementary File 1. Literature search strategy

**RESEARCH QUESTION**

Review of perioperative antimicrobial prophylaxis preventing infectious complications after radical cystectomy.

**PICO**

| **Population** | Patients treated with radical cystectomy |
| --- | --- |
| **Intervention** | Any empiric perioperative antimicrobial prophylaxis from procedure until discharge |
| **Comparison** | Protocols of different perioperative antimicrobial prophylaxis regimens |
| **Outcome** | Proportion of postoperative infectious complications, type and site of infection |

# **MEDLINE/ PUBMED - CONDUCTED SEARCH ON NOVEMBER 29^th^, 2021**

("cystectomy"[Title/Abstract] OR “cystectomy”[Mesh])

AND

("Postoperative Complications" [Mesh] OR "infection*"[Title/Abstract] OR "sepsis"[Title/Abstract] OR "sepsis" [Mesh])
AND 
(“antibiotic prophylaxis”[Mesh] OR “anti-infective agents”[Mesh] OR "antibiotic*"[Title/Abstract] OR "prophyla*"[Title/Abstract])

| Cystectomy | Postoperative complication | Antimicrobial Prophylaxis | Search results |
| --- | --- | --- | --- |
| X |  |  | 18,456 |
|  | X |  | 2,197,499 |
|  |  | X | 1,099,562 |
| X | X | X | 210 |

# **EMBASE - CONDUCTED SEARCH ON NOVEMBER 29^th^, 2021**

(cystectomy:ab,ti)

AND

('postoperative complications'/exp OR 'infection*':ab,ti OR 'sepsis':ab,ti OR 'sepsis'/exp)

AND

('antibiotic prophylaxis'/exp OR 'anti-infective agents'/exp OR 'antibiotic*':ab,ti)

NOT

('case report':ab,ti)

| Cystectomy | Postoperative complication | Antimicrobial Prophylaxis | Search results |
| --- | --- | --- | --- |
| X | X | X | 563 |

# **COCHRANE - CONDUCTED SEARCH ON NOVEMBER 29^th^, 2021**


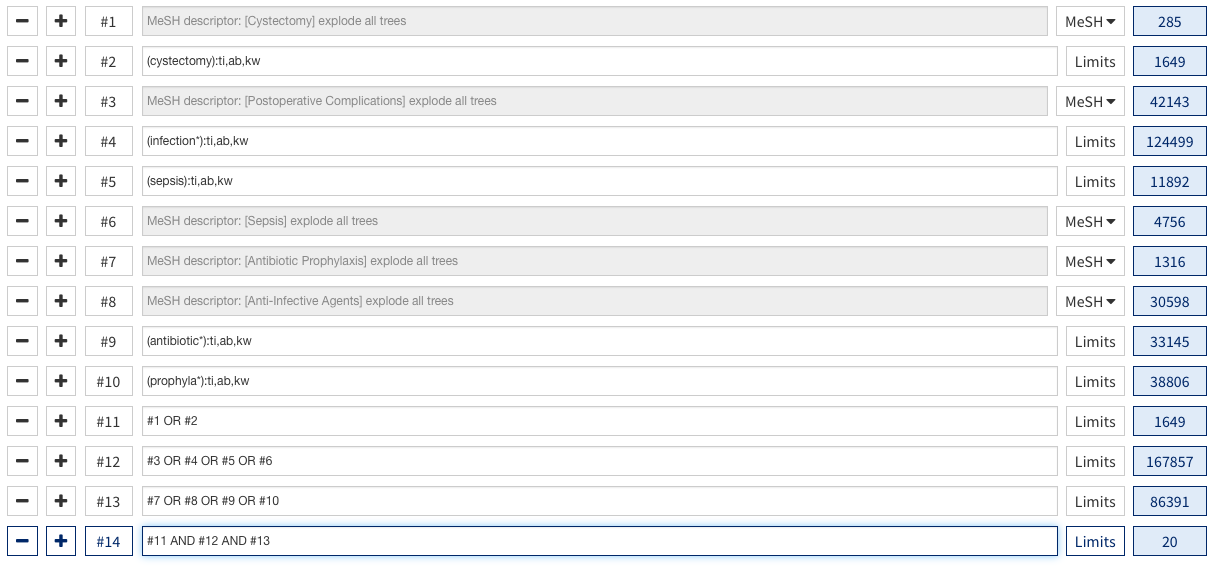


| Cystectomy | Postoperative complication | Antimicrobial Prophylaxis | Search results |
| --- | --- | --- | --- |
| X |  |  | 1,649 |
|  | X |  | 167,857 |
|  |  | X | 86,391 |
| X | X | X | 20 |
