# Supplementary material for: Association Between Antibiotic Prophylaxis Before Cystectomy or Stent Removal and Infection Complications: A Systematic Review
Source: Eur Urol Focus. Author manuscript; Available in PMC 2026 Apr 13. (PMC13071900; doi:10.1016/j.euf.2023.01.012)
Supplement: supplement 2 [file NIHMS2153973-supplement-supplement_2.docx]

**Detailed description of perioperative antibiotic management**

Empirical use of antibiotics for 1–3 days after surgery was described in 12 papers [8-19]. Hara and colleagues [9] used either 2 g IV piperacillin 2 hours after cystectomy or 2 g IV piperacillin every 12 hours for 3 days after surgery. Kim and colleagues [10] used IV cefotetan for their “short-term antibiotic” group of patients for the first post-operative day. Krasnow and colleagues [11] administered peri-operative antibiotics—primarily 1^st^/2^nd^/3^rd^ generation cephalosporins (80%), aminoglycosides (20%), and fluoroquinolones (20%)—for 1 or 2 days after surgery in 71% of patients. Nasu and colleagues [12] used IV ampicillin/sulbactam, cefazolin, or cefmetazole before the operation and continued use twice a day until post-operative day 2. Numao and colleagues [8] administered cefotiam hydrochloride until post-operative day 2 in 54 of 65 patients and until post-operative day 3 in 8 of 65 patients. Pariser and colleagues [13] used 2 g IV cefoxitin for 24 hours in 258 of 386 patients and 3 g ampicillin/sulbactam every 6 hours during surgery and added 4 mg/Kg gentamicin and 400 mg single-dose fluconazole for the next 24 hours in 128 of 386 patients. Ross and colleagues [14] administered IV antibiotics, mainly cephazolin/metronidazole (58%) or ampicillin/metronidazole/ciprofloxacin (30%) for 24 hours after surgery. Shigemura and colleagues [15] used 4.5 g piperacillin/tazobactam every 8 hours for 2 days after surgery. Takeyama and colleagues [16] used a different regimen of antibiotics for each patient until post-operative day 3, but they also administered pre-operative antibiotics if patients had bacteriuria before cystectomy. Tanaka and colleagues [17] administered 2.5 g piperacillin/tazobactam every 3 hours during surgery, then twice a day for 3 post-operative days. Wang and colleagues [18] used 2^nd^ generation cephalosporin for at least 3 days after surgery for 112 of 179 patients, prolonged only if a urine culture performed at post-operative day 3 was positive. Werntz and colleagues [19] used IV 2^nd^ generation cephalosporins for 24 hours after surgery in 50% of patients.

Empirical use of antibiotics for more than 3 days after surgery was described in 6 papers. Haider and colleagues [20] administered different regimens of antibiotics for a median of 7 days (IQR: 5–14). Wang and colleagues [18] used 2^nd^ generation cephalosporin for 67 of 179 patients for 7–10 post-operative days. Empirical use of antibiotics for more than 10 days was described in 4 papers. Kim and colleagues [10] used IV 3^rd^ generation cephalosporin for the “long-term antibiotic” group of patients for 10 post-operative days, as well as oral administration of either 3^rd^ generation cephalosporin or fluoroquinolones or trimethoprim/sulfamethoxazole for the subsequent 14 post-operative days. Kirkpatrick and colleagues [21] used different antibiotic regimens for a mean of 15 post-operative days. Goldberg and colleagues [22] administered different types of antibiotics during surgery, followed by either cefuroxime or ofloxacin until discharge. Werntz and colleagues [19] used IV 2^nd^ generation cephalosporins in 50% of patients for 30 post-operative days. Shigemura and colleagues [23] used different regimens of perioperative antibiotics for variable durations according to each doctor’s discretion.
