# Supplementary material for: Association Between Antibiotic Prophylaxis Before Cystectomy or Stent Removal and Infection Complications: A Systematic Review
Source: Eur Urol Focus. Author manuscript; Available in PMC 2026 Apr 13. (PMC13071900; doi:10.1016/j.euf.2023.01.012)
Supplement: supplement 3 [file NIHMS2153973-supplement-supplement_3.docx]

Supplementary Table 1. Quality assessment of the studies according to QUADAS-2

| References | Risk of bias | | | | Applicability concerns | | |
| --- | --- | --- | --- | --- | --- | --- | --- |
|  | Patient selection | Index test | Reference standard | Flow and timing | Patient selection | Index test | Reference standard |
| Beano | L | L | L | L | L | L | L |
| Goldberg | L | L | U | H | L | L | U |
| Haider | L | L | U | H | H | L | U |
| Hara | L | L | U | L | H | L | U |
| Kim2021 | H | L | U | H | L | L | U |
| Kim2018 | H | L | L | H | H | L | L |
| Kirkpatrick | L | L | U | L | L | L | U |
| Kolwijck | L | L | U | L | L | L | U |
| Krasnow | L | L | U | H | H | L | U |
| Nasu | L | L | L | L | L | L | L |
| Numao | H | L | L | L | L | L | L |
| Pariser | H | L | U | H | H | L | U |
| Ross | L | L | U | L | L | L | U |
| Shigemura2019 | L | L | U | L | L | L | U |
| Shigemura2012 | L | L | U | L | H | L | U |
| Takeyama | L | L | U | L | H | L | U |
| Tanaka | L | L | U | H | H | L | U |
| Van Horn | L | L | U | H | L | L | U |
| Wang | L | L | L | L | L | L | L |
| Werntz | H | L | U | L | L | L | U |

*L low risk of bias/applicability, H high risk of bias/applicability, U unclear risk of bias/applicability*

**Identification of studies via databases and registers**

Records removed *before screening*:

Duplicate records removed

(n = 119)

Records marked as ineligible by automation tools

(n = 0)

Records removed for other reasons

(n = 0)

Records identified from:

Pubmed (n = 210)

Embase (n = 563)

Cochrane Library (n = 20)

**Identification**

Records excluded

(n = 628)

Records screened

(n = 674)

Reports not retrieved

(n = 0)

Reports sought for retrieval

(n = 46)

**Screening**

Reports excluded:

General post-operative complications

(n = 9)

Lack of precise number of complications

(n = 5)

Lack of essential data

(n = 12)

Reports assessed for eligibility

(n = 46)

Studies included in review

(n = 20)

Reports of included studies

(n = 20)

**Included**

Supplementary Figure 1. Study flow chart

| ***Author*** | ***Year of publication*** | ***Institution*** *(Monocentric/*  *Multicentric)* | ***Study design*** *(Retrospective/*  *Prospective)* | ***Total number of patients*** |
| --- | --- | --- | --- | --- |
| **Beano et al.[1]** | 2019 |  | Prospective | 146 |
| **Goldberg et al.[2]** | 2018 | Monocentric | Retrospective | 405 |
| **Haider et al.[3]** | 2019 | Multicentric | Retrospective | 217 |
| **Hara et al.[4]** | 2008 | Monocentric | Retrospective | 77 |
| **Kim et al.[5]** | 2021 | Monocentric | Prospective | 92 |
| **Kim et al.[6]** | 2018 | Monocentric | Retrospective | 287 |
| **Kirkpatrick et al.[7]** | 2018 | Monocentric | Retrospective | 75 |
| **Kolwijck et al.[8]** | 2019 | Monocentric | Retrospective | 147 |
| **Krasnow et al.[9]** | 2017 | Multicentric | Retrospective | 52349 |
| **Nasu et al.[10]** | 2017 | Monocentric | Retrospective | 50 |
| **Numao et al.[11]** | 2020 | Monocentric | Retrospective | 123 |
| **Pariser et al.[12]** | 2016 | Monocentric | Retrospective | 386 |
| **Ross et al.[13]** | 2021 | Monocentric | Retrospective | 165 |
| **Shigemura et al.[14]** | 2019 | Multicentric | Prospective | 49 |
| **Shigemura et al.[15]** | 2012 | Multicentric | Retrospective | 57 |
| **Takeyama et al.[16]** | 2005 | Monocentric | Prospective | 104 |
| **Tanaka et al.[17]** | 2012 | Monocentric | Prospective | 35 |
| **VanHorn et al.[18]** | 2018 | Monocentric | Retrospective | 279 |
| **Wang et al.[19]** | 2021 | Multicentric | Retrospective | 179 |
| **Werntz et al.[20]** | 2018 | Multicentric | Retrospective | 84 |

Supplementary Table 2. Baseline characteristics of included studies

1. Beano, H., *Lasix renal scintigraphy results correlate with ureteroenteric stricture rates and total readmission rates following radical cystectomy.* The Journal of Urology, 2019: p. Vol. 201, No. 4S.

2. Goldberg, H., et al., *Predictors of surgical site infection after radical cystectomy: should we enhance surgical antibiotic prophylaxis?* World J Urol, 2019. **37**(6): p. 1137-1143.

3. Haider, M., et al., *Use and duration of antibiotic prophylaxis and the rate of urinary tract infection after radical cystectomy for bladder cancer: Results of a multicentric series.* Urol Oncol, 2019. **37**(5): p. 300 e9-300 e15.

4. Hara, N., et al., *Perioperative antibiotics in radical cystectomy with ileal conduit urinary diversion: efficacy and risk of antimicrobial prophylaxis on the operation day alone.* Int J Urol, 2008. **15**(6): p. 511-5.

5. Kim, A.H., et al., *Novel Antibiotic-Irrigating Wound Protector Reduces Infectious Complications in Robot-Assisted Radical Cystectomy with Extracorporeal Urinary Diversion.* Urology, 2022. **159**: p. 160-166.

6. Kim, C.J., et al., *Impact of a change in duration of prophylactic antibiotics on infectious complications after radical cystectomy with a neobladder.* Medicine (Baltimore), 2018. **97**(47): p. e13196.

7. Kirkpatrick, C., A. Haynes, and P. Sharma, *Antibiotic prophylaxis is not associated with reduced urinary tract infection-related complications after cystectomy and ileal conduit.* Bladder (San Franc), 2018. **5**(3): p. e35.

8. Kolwijck, E., et al., *Incidence and microbiology of post-operative infections after radical cystectomy and ureteral stent removal; a retrospective cohort study.* BMC Infect Dis, 2019. **19**(1): p. 303.

9. Krasnow, R.E., et al., *Prophylactic Antibiotics and Postoperative Complications of Radical Cystectomy: A Population Based Analysis in the United States.* J Urol, 2017. **198**(2): p. 297-304.

10. Nasu, Y., *Clinical study on the preventative efficacy of culture-based targeted antibiotic prophylaxis for febrile urinary tract infections after removal of ureteral stents in radical cystectomy patients with urinary diversion.* Jpn. J. Chemother, 2017: p. 65 (6): 806-811.

11. Numao, N., et al., *Intraoperative Only versus Extended Duration Use of Antimicrobial Prophylaxis for Infectious Complications in Radical Cystectomy with Intestinal Urinary Diversion.* Urol Int, 2020. **104**(11-12): p. 954-959.

12. Pariser, J.J., et al., *The effect of broader, directed antimicrobial prophylaxis including fungal coverage on perioperative infectious complications after radical cystectomy.* Urol Oncol, 2016. **34**(3): p. 121 e9-14.

13. Ross, J.P.J., et al., *Association between radical cystectomy prophylactic antimicrobial regimen and postoperative infection.* Can Urol Assoc J, 2021. **15**(12): p. E644-E651.

14. Shigemura, K., et al., *Efficacy of Prophylactic Antimicrobial Administration of Tazobactam/Piperacillin for Radical Cystectomy with Urinary Diversion: A Multicenter Study.* Urol Int, 2019. **102**(3): p. 293-298.

15. Shigemura, K., et al., *Post-operative infection and prophylactic antibiotic administration after radical cystectomy with orthotopic neobladder urinary diversion.* J Infect Chemother, 2012. **18**(4): p. 479-84.

16. Takeyama, K., et al., *Incidence of and risk factors for surgical site infection in patients with radical cystectomy with urinary diversion.* J Infect Chemother, 2005. **11**(4): p. 177-81.

17. Tanaka, K., et al., *Analysis of isolated bacteria and short-term antimicrobial prophylaxis with tazobactam-piperacillin (1:4 ratio) for prevention of postoperative infections after radical cystectomy.* J Infect Chemother, 2012. **18**(2): p. 175-9.

18. VanHorn, C., *Urinary tract infection following radical cystectomy with an enhanced recovery protocol.* The Journal of Urology, 2018: p. Vol. 199, No. 4S.

19. Wang, Y., et al., *A perioperative management to reduce rate of urinary tract infection for patient underwent radical cystectomy with ileal conduit diversion.* Int Urol Nephrol, 2021. **53**(3): p. 401-407.

20. Werntz, R.P., et al., *Prophylactic antibiotics following radical cystectomy reduces urinary tract infections and readmission for sepsis from a urinary source.* Urol Oncol, 2018. **36**(5): p. 238 e1-238 e5.
